# Spatioselective surface chemistry for the production of functional and chemically anisotropic nanocellulose colloids

**DOI:** 10.1039/d2ta05277f

**Published:** 2022-11-03

**Authors:** Katja Heise, Tetyana Koso, Alistair W. T. King, Tiina Nypelö, Paavo Penttilä, Blaise L. Tardy, Marco Beaumont

**Affiliations:** Department of Bioproducts and Biosystems, Aalto University P.O. Box 16300 FI-00076 Aalto Espoo Finland; Materials Chemistry Division, Chemistry Department, University of Helsinki FI-00560 Helsinki Finland; VTT Technical Research Centre of Finland Ltd., Biomaterial Processing and Products 02044 Espoo Finland; Chalmers University of Technology 41296 Gothenburg Sweden; Wallenberg Wood Science Center, Chalmers University of Technology 41296 Gothenburg Sweden; Khalifa University, Department of Chemical Engineering Abu Dhabi United Arab Emirates; Center for Membrane and Advanced Water Technology, Khalifa University Abu Dhabi United Arab Emirates; Research and Innovation Center on CO2 and Hydrogen, Khalifa University Abu Dhabi United Arab Emirates; Institute of Chemistry of Renewable Resources, Department of Chemistry, University of Natural Resources and Life Sciences Vienna (BOKU), Konrad-Lorenz-Str. 24 A-3430 Tulln Austria marco.beaumont@boku.ac.at

## Abstract

Maximizing the benefits of nanomaterials from biomass requires unique considerations associated with their native chemical and physical structure. Both cellulose nanofibrils and nanocrystals are extracted from cellulose fibers *via* a top–down approach and have significantly advanced materials chemistry and set new benchmarks in the last decade. One major challenge has been to prepare defined and selectively modified nanocelluloses, which would, *e.g.*, allow optimal particle interactions and thereby further improve the properties of processed materials. At the molecular and crystallite level, the surface of nanocelluloses offers an alternating chemical structure and functional groups of different reactivity, enabling straightforward avenues towards chemically anisotropic and molecularly patterned nanoparticles *via* spatioselective chemical modification. In this review, we will explain the influence and role of the multiscale hierarchy of cellulose fibers in chemical modifications, and critically discuss recent advances in selective surface chemistry of nanocelluloses. Finally, we will demonstrate the potential of those chemically anisotropic nanocelluloses in materials science and discuss challenges and opportunities in this field.

## Abstract 2

In this review, we explain the influence and role of the multiscale hierarchy of cellulose fibers in their chemical modifications as exemplified through recent advances in the spatioselective surface chemistry of nanocelluloses.

## Graphical abstract

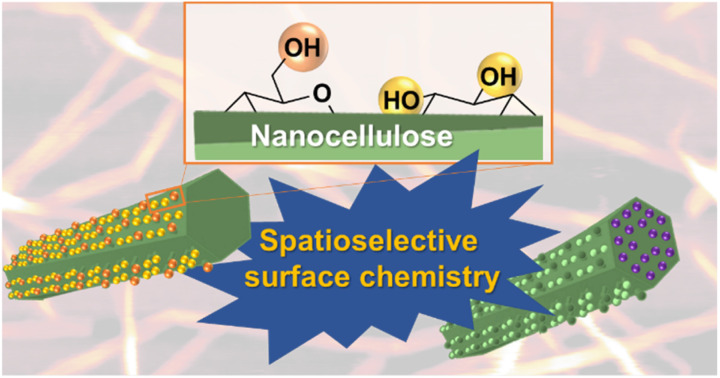

## 1. Introduction

**Fig. 1:**
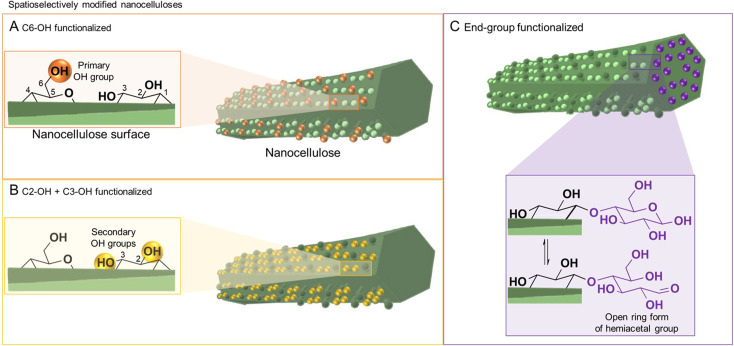
Nanocelluloses, *i.e.*, cellulose nanofibrils and cellulose nanocrystals, feature a native anisotropic chemical structure due to the presence of surface functional groups of varying reactivity at their surface (A and B) and end (C). This offers a unique opportunity to modify selectively either at the positions of C6–OH (orange balls, A) or C2–, C3–OHs (yellow balls, B); and the reducing end-group (highlighted in purple, C); giving straightforward access to spatioselectively modified nanoparticles. The reducing end-group is in an equilibrium of the unreactive ring and the reactive chain form bearing an aldehyde group (inset in C).

The utilization of bio-based colloids in materials science has witnessed exponential growth during the past decade,^1^ which has been largely amplified by the climate crisis,^2^ hazardous issues associated with the lifecycle of synthetic thermoplastics,^3^ and the global urge to shift to a bio-based economy. This renders the development of renewable functional nanomaterials and sustainable materials chemistry most timely and necessary. In this context colloids, such as cellulose nanocrystals and cellulose nanofibrils, are at the forefront of materials science. Both are based on the structural building blocks of wood cell walls, *i.e.* the elementary fibrils (also called microfibrils), which have a diameter of approx. 2–4 nm (Fig. 1) and extremely high tensile strength and modulus of up to 7 GPa^4,5^ and 140 GPa,^6–8^ respectively. This exceeds the corresponding properties of most metals, synthetic polymers, and even many ceramics (especially considering the low cellulose density),^5^ and explains the strong and rising interest in nanocellulose-based materials.

In this review, we will explain the influence and role of the multiscale hierarchy of cellulose fibers in chemical modifications, critically discuss recent advances in selective surface chemistry of nanocelluloses and demonstrate the potential of those chemically anisotropic nanocelluloses in materials science.

Cellulose has three available surface hydroxyl groups, the primary C6–OH as well as the two secondary C2–OH and C3–OH (Fig. 1A and B) with each having specific reactivity. Besides bearing accessible hydroxyls, nanocelluloses have aldehyde (hemiacetal) groups – the so-called reducing end-groups (REGs) – on one side of the polymer chain, which is in the case of cellulose nanocrystals located at the end of the particle (Fig. 1C). The functional groups can be selectively reacted to yield chemically anisotropic nanocelluloses, *e.g*., C6–OH– (Fig. 1A), C2–OH and C3–OH– (Fig. 1B), or end group-functionalized nanocelluloses (Fig. 1C). These chemical anisotropies at the nanocellulose surface represent a hitherto barely exploited potential in materials chemistry, enabling highly selective molecular patterning to finetune the surface chemistry and interactions of these nanoparticles.

**Fig. 2:**
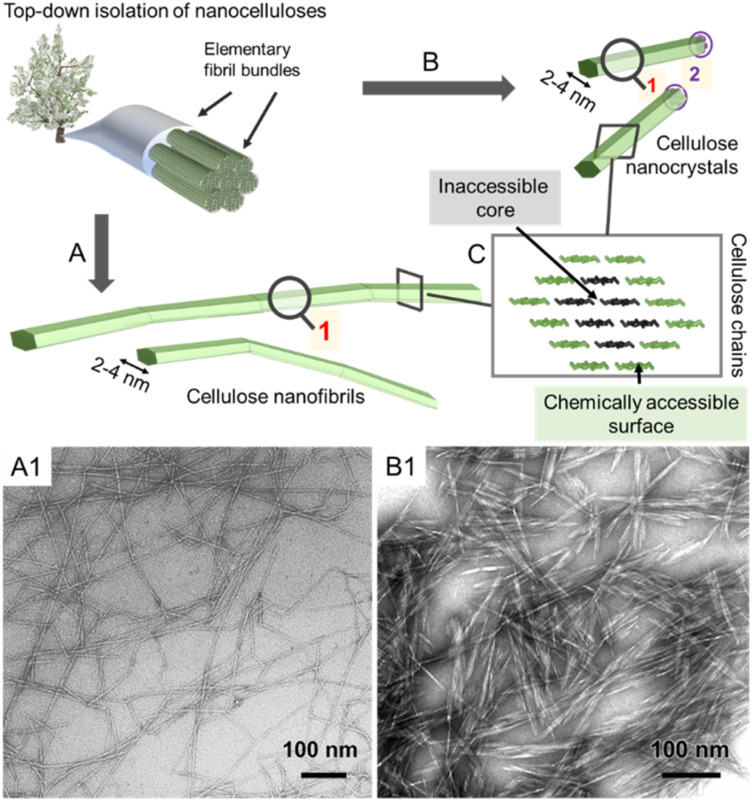
Simplified schematic of the hierarchically structured wood-based cellulose fiber composed of individual elementary fibrils, *i.e.*, microfibrils, can be either deconstructed into cellulose nanofibrils (A) or cellulose nanocrystals (B). The microfibrils, as well as the individual CNFs and CNCs, feature a chemically accessible surface and non-accessible core (C). The transmission electron micrographs of CNFs and CNCs are shown in A_1_ and B_1_, respectively. (A_1_) and (B_1_) were adapted with permission from Saito *et al.*^11^ and Xu *et al.*^12^ Copyright 2007 and 2013 American Chemical Society.

All reactions, either in fiber or nanoparticle scale, occur at a solid interface, the respective particle surface, and it is important to emphasize that typical nanocelluloses are produced *via* a top–down approach, from the cellulose fiber into a nanoparticle (Fig. 2). This is in contrast to most organic nanoparticles, which are generally assembled *via* a bottom–up approach from defined and soluble synthetic precursors, *i.e*., polymers or monomers. Despite the relatively simple chemical structure of cellulose chains based on β-O-1,4-linked glucopyranose repeating units,^9^ the structure of the cellulose fiber is complex and composed of hierarchically ordered rigid elementary fibrils (Fig. 2). We use cellulose fiber as a term to describe a processed fiber of high cellulose content.

The elementary fibrils represent the smallest particle subunit (further information in Section 2). During deconstruction into nanocellulose, the cellulose fiber is broken down into bundles of elementary fibrils (Fig. 2A, cellulose nanofibers with a diameter below 100 nm), individual cellulose nanofibrils (CNFs, 2–4 nm diameter), or into shorter, more crystalline cellulose nanocrystals (Fig. 2B, CNCs).^1,10^ Here, we will focus on the rather defined, individualized nanocelluloses, CNFs and CNCs.

The size and reactivity of nanocelluloses are ultimately dictated by the native hierarchical structure of the cellulose fiber, *e.g.*, the size of the microfibril (Section 2), the composition of the cellulose fiber, and the used chemical treatments during the deconstruction into nanocelluloses (Section 3.1). In the latter sections, we introduce the current state of the art in surface chemistry of nanocelluloses and add the perspective of regioselectivity (Section 3.2–3.4). Finally, we associate the impact of regioselectivity with the possibility to tune and control the properties of functional nanocelluloses (Section 4) for designing the next-generation cellulosic nanomaterials.

## 2. Cellulose microfibril structure and reactivity

Cellulose is produced in nature by plants and other organisms, including some bacteria and animals. It is synthesized by cellulose-synthesizing complexes (CSCs), which are enzyme complexes that polymerize hundreds or thousands of uridine diphosphate–glucose units into β-1,4-linked glucan chains.^13^ The newly formed cellulose chains are extruded into the medium surrounding the cell membrane, in which they form semicrystalline aggregates called microfibrils (or elementary fibrils). Due to a rotation of the glycosidic linkage for each glucose monomer unit, the chain obtains a two-fold helical symmetry leading to alternating functionalities of C6–OH, and C2– and C3–OH groups on the microfibril surface (Fig. 1A and B).^14^ Cellulose in nature mostly exists in the cellulose I crystal structure, whereas cellulose II is formed upon mercerization or regeneration from dissolved cellulose.^15^ Cellulose II is characterized by the anti-parallel arrangement of the cellulose chains.^16^ In the native cellulose I, which exists in two forms called cellulose Iα and Iβ, parallel chains are packed in layers with the pyranose rings oriented in the plane.^17,18^ These layers are held together by hydrogen bonding and a smaller contribution from London dispersion forces (a type of van der Waals forces), whereas the stacking of the layers is dominated by the dispersion forces.^19,20^ The morphology of the microfibrils directly affects the hydrophobicity and hydrophilicity of the crystallite surfaces and the chemical groups available for modification.

**Fig. 3:**
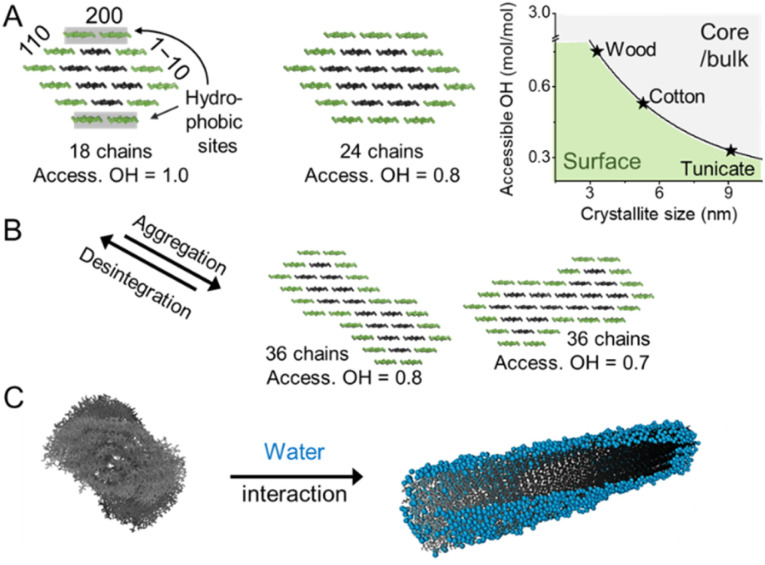
(A) Cellulose chains assemble in the form of microfibrils, shown exemplarily in the form of the 18- and 24-chain models. The microfibril size defined by the chain arrangement and number dictates the relative amount of accessible hydroxyl groups at the microfibril surface (access. OH in mol mol^−1^). (B) Interfibrillar interactions between microfibrils, *e.g*., in a microfibril bundle, may lead to aggregation decreasing the available OH at the surface and increasing the crystallinity. The disintegration of such aggregates, *e.g.*, during the preparation of CNFs exposes OH-groups and decreases sample crystallinity. The chain arrangements in the microfibrils expose hydrophobic (200) and hydrophilic (110, 1–10) crystal surfaces and water molecules interact preferably with hydrophilic crystal surfaces, as shown in the modeled structure (C). (C) was adapted with permission from Paajanen *et al.*^27^ Copyright 2019 Springer Nature. The plot in A was drawn according to Okita *et al.*^34^

The exact shape of the wood microfibril cross-section has not been fully verified yet, whereas the most plausible candidates are based on an 18,^21,22^ or 24-chain model (Fig. 3A).^23–25^ The most common models of an average microfibril expose crystal surfaces with both hydrophobic (200 in cellulose Iβ) and hydrophilic characters (1–10 and 110 in cellulose Iβ). Furthermore, the currently most promising candidate for the 18-chain microfibril, *i.e.* one with 2, 3, 4, 4, 3, and 2 chains in the layers, offers two possible arrangements corresponding to different proportions of the 1–10 and 110 surfaces (one of which is shown in Fig. 3A).^26^ The interaction of water molecules shown in Fig. 3C is hence selective to the hydrophilic 1–10 and 110 planes,^27^ and nanocelluloses are considered amphiphilic particles.^28^ The crystalline arrangement of cellulose chains in the microfibril is susceptible to slight modifications for instance due to mechanical stresses and interactions with moisture.^29–31^ More drastic changes in the microfibril structure may be induced by partially irreversible aggregation/co-crystallization of microfibrils, *e.g.*, during drying at high temperatures, or disintegration and partial decrystallization of microfibril aggregates during the deconstruction of the cellulose fiber into CNFs (Fig. 3B).^26,32,33^ Therefore, the shape of a cellulose microfibril cross-section and the number of chains forming it may vary due to both biological reasons and the processing history of the material.

**Fig. 4:**
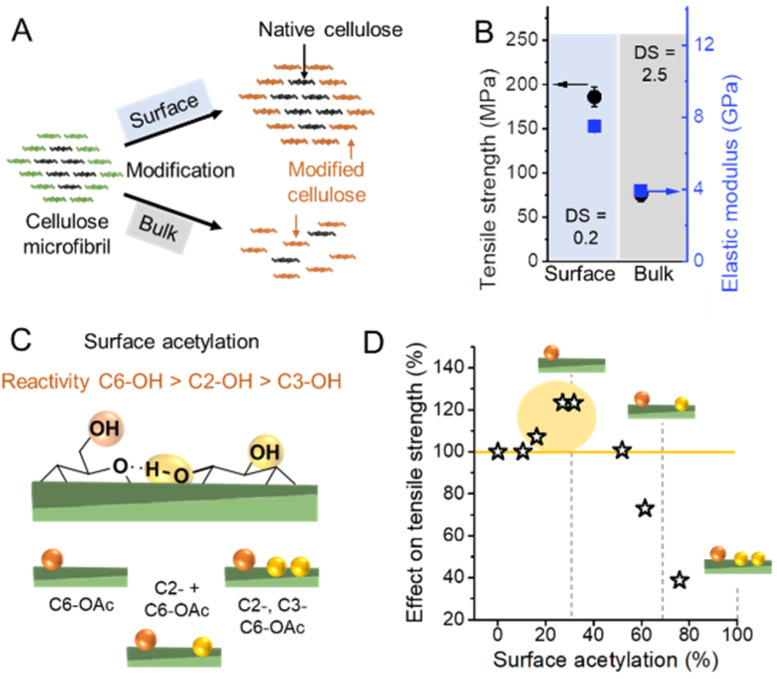
(A) Schematic representation of a surface *vs.* bulk modification. The microfibril consists of chemically accessible surface chains (green color) and inaccessible chains in the core (black). Surface modification is confined to the surface, whereas bulk modification also affects the cellulose chains in the non-accessible, more crystalline core causing ultimately an alteration of the microfibril structure at a high degree of substitution. (B) Influence on mechanical properties of surface^53^*vs.* bulk acetylation.^54^ (C) Possible combination of acetyl group substitution patterns at the surface of cellulose. (D) Influences of the extent of surface acetylation on the mechanical properties of cellulose. Plot B was drawn from literature data by Beaumont *et al.*^53^ and Cindradewi *et al.*,^54^ and Plot D from data by Aiken *et al.*^47^

Due to the small crystal size and the high ratio of surface to inner chains in the microfibrils of land plants, their degree of crystallinity is relatively low. Here the degree of crystallinity should be understood as an indicator of the degree of order in the crystallites, which is different from synthetic polymers exhibiting separated amorphous and crystalline domains. In this regard, the plant cell wall may be regarded as meso-crystalline, with regions where there are varying degrees of disorder. In general, the cellulose chains located at the surface are chemically accessible and can be considered to be less crystalline, *i.e*., less ordered, whereas the chains in the microfibril core are chemically inaccessible and more crystalline, *i.e.*, more ordered (Fig. 2). Heterogeneous reactions or surface reactions of cellulose are hence confined to the surface hydroxyl groups.^1^ Larger crystallites are produced, *e.g.*, by algae,^35^ cotton,^36^ and bacteria,^37^ and give access to nanocelluloses of bigger fibril diameter and different aspect ratios.^38,39^ As shown in Fig. 3A, the amount of accessible OHs decreases with increasing crystallite size, simply because the relative number of surface chains is reduced. The size and structure of the microfibril are crucial to predict the number of accessible hydroxyl groups (Fig. 3A and B). In the case of wood-derived cellulose (18 or 24 chains per microfibril), 25–33% of the total hydroxyl groups in the microfibril are at the surface and hence chemically accessible, which corresponds to a degree of substitution (DS) of 0.75–1.00 (total amount of OH-groups per monomer unit is 3, the maximum DS is 3). In comparison, aggregated cellulose microfibrils, *e.g.*, in a fiber construct (Fig. 2), feature a lower reactivity as fewer hydroxyl groups are available at the surface (Fig. 3B). If a reaction proceeds into the core (containing inaccessible OH groups) of the microfibril, for instance, due to harsher reaction conditions, it can be categorized as a bulk modification. Such modification alters the physical properties of cellulose, such as transparency, and mechanical or thermal properties.^40^ This is exemplarily shown for acetylated cellulose in Fig. 4B, comparing the tensile strength and elastic modulus of a surface-acetylated sample (DS = 0.2) with a highly substituted cellulose acetate (DS = 2.5), the elastic modulus and tensile strength are significantly reduced in the case of the cellulose acetate sample. Consequently, chemical modification in the course of the production of functional nanocelluloses should be confined to the surface, to preserve their intrinsic properties.

The reactivity of the hydroxyl groups decreases in the following order, C6–OH > C2–OH > C3–OH, although all three hydroxyl groups are in theory accessible at the surface of cellulose fibrils. Experiments have shown that the C3–OH is hardly reactive due to steric effects and intrachain hydrogen bonding.^41–44^ Consequently, the DS threshold for surface modifications (Fig. 3A), such as acetylation, is lower as only C6–OH and C2–OH are chemically accessible under heterogeneous conditions. This is also supported by the literature that discusses an upper esterification limit of approximately 66% in surface esterifications.^43,45,46^

Hence the extent of a surface modification largely influences the mechanical properties of celluloses (Fig. 4B and D), and the influence of the modification on the properties has to be constantly monitored to reach an optimal level, even at relatively low DS values.^47^ Common acetylation of cellulose as shown in Fig. 4C is not regioselective, however, one can assume that until surface acetylation of 30–40% most acetylation occurs at the primary C6–OH due to its higher reactivity. An improvement in mechanical properties for C6–OH modified samples has been also shown in the case of CNFs.^48^ A surface DS above 40% and hence modifications at C2–OH and C3–OH have been shown to reduce significantly the tensile strength.

This can be most probably attributed to a partial modification of C_3_–OH, which will ultimately affect the crystalline domains and cause polymer degradation and occur below the theoretical limit of surface acetylation, thereby diminishing mechanical properties.^49,50^ Similar observations were also made in the esterification of CNFs and CNCs, which confirm that a low DS (even below the limit of surface modification) is recommended to avoid fragmentation and degradation of nanocelluloses.^51,52^ Controlling the extent of reaction and surface-confinement is hence of utmost importance, and can be achieved through comparison of crystallinity before and after modification,^42^ but is still not very common in the literature.

Solvent interactions of cellulose particles influence their dispersibility, swelling, and chemical accessibility in a given solvent, and are thereby also a crucial aspect influencing the cellulose reactivity. The abundance of hydrophilic surface hydroxyl groups in cellulose explains its hygroscopicity and sorption-induced swelling.^55,56^ The main contributors to the water interactions are the surfaces of the microfibrils,^57,58^ that are covered with a layer of water, under ambient conditions (Fig. 3C).^27,55^ Due to the strong cellulose–water (or hemicellulose–water) interactions, this surface-bound or otherwise spatially confined water can be distinguished from bulk water by its properties and is referred to as bound water.^59–62^ Unprocessed, native wood cell walls swell by water adsorption on the crystallite surfaces and in the hemicelluloses separating the cellulose microfibrils.^30^ Similarly, swelling in processed celluloses may be attributed to residual hemicelluloses or interfacial disorder between neighboring microfibrils. Such disorder in pure celluloses could be caused by a mismatch of the crystallite orientations, for instance, due to twisting.^27^ From the viewpoint of chemical modification in aqueous environments, the location of water in the cellulose nanostructure and its interactions with the microfibril bundles are crucial. The swelling of the microfibril bundles and the size of water clusters, the connectivity of their network, and the width of water channels control the accessibility of soluble chemical agents to the lower levels of the hierarchical structure. All of these are affected by the moisture content and the origin and processing history of the cellulosic sample, which modify the aggregation state and crystallinity of the cellulose microfibrils. The amount of confined water in the fiber structure is especially important in solid-state^43^ or gas-phase reaction^63^ and will be further discussed in Section 3.3.

## 3. Preparation of functional nanocelluloses

In this section, we will first introduce general methods to isolate CNFs and CNCs from the fiber source (Section 3.1) and basic avenues to produce chemically functionalized nanocelluloses (Section 3.2). We further introduce and discuss in detail pathways toward regioselectively modified nanocelluloses, which ultimately enable in a controlled manner spatially confined modification at the nanoparticle surface (Section 3.3). An overview of analytical methods to characterize those functional nanocelluloses is presented in Section 3.4.

### 3.1. Deconstruction: from fibers to nanocelluloses

**Fig. 5:**
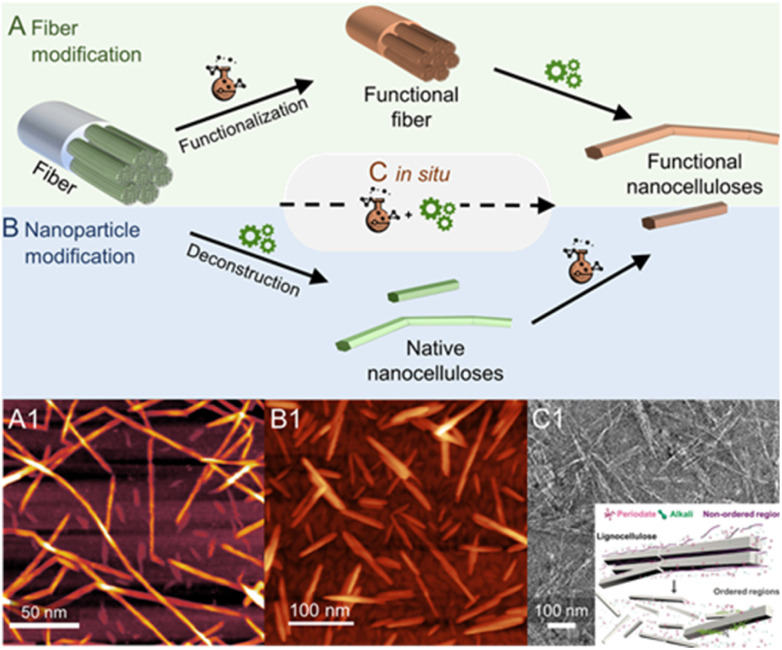
Most frequently used chemical pathways to functional nanocelluloses from cellulose fibers. Regioselective modification of cellulose is either conducted (A) before, (B) after or (C) *in situ* during the deconstruction of the fiber into cellulose nanocrystals (CNCs) or nanofibrils (CNFs). In general, the deconstruction is either a chemical process, *e.g.*, the acidic hydrolysis into individual CNCs, or a mechanical approach in the case of fibrillation yielding CNFs. Shown micrograph examples are, (A_1_) succinylated CNFs prepared *via* pathway A, (B_1_) acetylated CNCs prepared from native CNCs (pathway B), and (C_1_) the *in situ* production of CNCs bearing carboxylate groups directly from cellulose fibers *via* periodate oxidation. The latter combines both functionalization and deconstruction in a single step (C). (A_1_) was adapted with permission from Beaumont *et al.*^90^ under CC BY 4.0. Copyright 2021 American Chemical Society. (B_1_) was reproduced from Koso *et al.*^44^ with permission from the Royal Society of Chemistry. (C_1_) was adapted with permission from Liu *et al.*^91^ under CC BY 4.0. Copyright 2020 Wiley.

The production of nanocelluloses, *i.e*. CNF and CNCs, has been well reviewed^1,64–66^ and will, therefore, be only briefly discussed. In general, completely individualized CNFs are produced by a chemical pretreatment of the cellulose fiber, modifying the fiber surface (Fig. 5A_1_), and subsequent mechanical fibrillation. The purpose of the modification step is to decrease interfibrillar interactions, either through the introduction of repulsive ionic charges (*e.g*., carboxylate or quarternary ammonium groups) or in general by decreasing the number of available hydroxyl groups for hydrogen bonding (*e.g.*, aliphatic ester groups).^1^ Consequently, a controlled modification significantly reduces the energy required for mechanical fibrillation, which is usually conducted *via* high-pressure homogenization,^67–69^ microfluidization,^70–72^ or high-intensity ultrasonication,^73,74^ and facilitates the deconstruction of the fiber into individualized cellulose microfibrils. In addition, from a practical viewpoint, the chemical modification at the fiber level has the advantage of a more straightforward and efficient purification, since remaining reactants or solvents can be easily removed by normal centrifugation or filtration methods, and reactions can be conducted at high consistency to improve their efficiency.^75^ What we obtain through this combined isolation pathway, however, is essentially a cellulose derivative, at least on the nanofibril surface, which has properties that significantly differ from that of non-modified cellulose.

Similar to CNFs, the introduction of repulsive charges facilitates the isolation of individualized CNCs and provides them with their unique colloidal properties. The different production pathways and sources are reviewed in detail.^76–78^ The most common pathway affording the rod-shaped nanocrystals is hydrolysis in 64-wt% sulfuric acid, which introduces sulfate half-ester (–OSO_3_^−^) groups to the nanocrystal surface through esterification. Alternatively, aqueous HCl^79,80^ or HBr^81,82^ have been used. However, the resulting non-charged nanocrystals have poor dispersibility in water. Gaseous HCl also affords CNCs at a high yield (>80%) and has been combined with a subsequent TEMPO-oxidation and high-intensity ultrasonication to sufficiently disintegrate and disperse the hydrolyzed material.^83^*In situ* introduction of charges to the CNC surface during the top–down isolation, similarly to the H_2_SO_4_ pathway but far less common, can be also achieved with phosphoric acid^84–86^ or through hydrolysis with organic acids (*e.g*., oxalic or formic acid)^87,88^ esterifying the nanocrystal surface with anionic –OPO_3_^−^ or –COR groups (where *R* is any substituent), respectively. Ultrasonication or high-pressure fluidization is in all cases an indispensable tool to ensure complete individualization of the CNCs and to disintegrate potential nanocrystal aggregates after acid hydrolysis. An interesting pathway toward functionalized CNCs is the combination of acid hydrolysis and Fischer esterification, which has been used to attach a whole range of functionalities, from polymerization initiators, to double or triple bonds, to the CNC surface *in situ* during its production.^89^

### 3.2. Preparation of functional nanocelluloses

Nanocelluloses decorated with chemical moieties, *e.g.*, ester or oxidized groups can be obtained by conducting surface chemistry on a micrometer or nanometer-sized particles, *i.e.*, the cellulose fiber or the native nanocellulose.^1^ Hence, the chemical modification is conducted either before (fiber modification, Fig. 5A)^48,53,90^ or after the deconstruction of the fiber into a colloidal state (nanoparticle modification, Fig. 5B).^44^ Alternatively, there have been also endeavors to isolate modified nanocellulose directly from fibers or even raw biomass (Fig. 5C and C_1_), by a combination of chemical modification and deconstruction in a single step.^91–93^ Choosing the order of processing steps is highly dependent on the used starting material, the targeted functionality, and the type of nanocellulose, *e.g.*, modifications of CNCs are often conducted starting from dried CNCs (Fig. 5B) since CNCs can be dispersed from the dry state due to their morphology. In addition, many functionalities, *e.g.*, ester groups, would be removed during the acidic hydrolysis step of CNC production.

Modification of CNFs with the same pathway (nanoparticle modification) is conducted in dispersion with low solid content (mostly below 5 wt%), which renders most chemical modifications inefficient. The reactions are generally conducted in a never-dried state, since the large aspect ratio of CNFs and their high cohesive interactions, cause the formation of irreversible aggregates upon drying or solvent removal.^94^ CNF reactions are either conducted directly in aqueous dispersion,^95,96^ in which water can be disturbing as it might react with the used reactants (*e.g.*, anhydride, acid chlorides), or in organic solvents, which require a solvent exchange.^97^ Taking this into account, the fiber modification (Fig. 5A) is often selected for the preparation of functional CNFs,^11,53,90^ since it can be conducted at higher solid content to increase the reaction efficiency,^75^ and offers a more straight-forward purification step due to the larger fiber size, which does not require ultrafine membrane filtration or dialysis steps.

The composition of the used cellulose is important to consider since residual non-cellulosic biopolymers can influence the chemical modification,^48^ or adsorb onto colloids influencing greatly their properties,^98–101^ and reactivity.^48^ Further details on the extraction of cellulose from wood^1,102^ and other sources^103^ are accessible in the literature. For the simple reason of simplification and increasing the control of chemical reactions, most modifications of (nano)celluloses are performed on cellulose fibers (>90% cellulose purity),^90,95,102,104^ so-called dissolving pulp, which contrast with raw or paper-grade cellulosic fibers containing significant amounts of lignin and hemicelluloses.

### 3.3. Regioselective modification of nanocelluloses

We encompass in this discussion selective chemical modifications at a certain type of surface hydroxyl groups (primary C6– *vs.* secondary C2–, C3–OHs, Fig. 1A and B, Sections 3.2.1 and 3.2.2) or the reducing end-group (Fig. 1C, Section 3.2.3). The focus lies on methods, which are straightforward, mild, and have none or only minor influences on the physical properties of nanocelluloses (crystallinity, molar mass, *etc.*); and are hence suitable for their functionalization. This stays in contrast to traditional chemical pathways toward regioselectively modified celluloses,^105–107^ which are not surface-confined, require multi-step syntheses routes including protective groups (these groups are priorly introduced to ensure modification at a specific, free hydroxyl group), and/or are conducted under harsh conditions.

Concerning surface hydroxyl group modification, we distinguish between different reaction classes, (a) esterifications as a reversible chemical substitution of the cellulose backbone (Section 3.2.1, schemes of esterified nanocelluloses are colored in blue), and (b) oxidations, which are irreversible and change the chemical structure of the cellulose backbone at the fibril surface (Section 3.2.2, schemes of oxidized nanocelluloses are colored in orange or yellow).

#### 3.3.1 Esterifications (C6–OH selectivity)

Esterification and mostly acetylation are one of the most successful commercial reactions to produce cellulose-based chemicals. Traditionally, it is performed under heterogeneous conditions using acetic anhydride in acetic acid, with sulphuric acid as catalyst and it is a very simple method to modify free hydroxyl groups on cellulose surfaces and, thus, reduce hydrogen-bonding.^108–110^ This reaction itself is not regioselective, but in general follows the order of reactivity of the hydroxyl groups, as detailed in Section 2. Such reactions might be at the beginning more preferable at C6–OH, but acetylation of C2–OH or C3–OH is likely to occur to a certain degree in parallel, and the reaction proceeds into the bulk/crystalline regions of the cellulose.^111^ In the industry, the focus still lies on the fabrication of cellulose with very high or complete bulk acetylation. Bulk acetylation completely changes the native microfibril structure and properties, and hence those esterification methods are not suitable for CNFs or CNCs, where crystallinity and morphology should be preserved.

If the traditional H_2_SO_4_-catalysed acetylation conditions are applied to CNCs, practically all the original cellulose I crystallinity is removed and acetylation DS values reach approx. 2.^112^ This process yields also lower DS acetylated CNCs of cellulose II crystal structure, which have a rod-like shape.^113^ As acetylation beyond surface modification causes dissolution of cellulose, this phenomenon can be also beneficial, *e.g.*, to extract acetylated cellulose nanofibers in an energy-efficient manner from a swollen, partially dissolved cellulose matrix.^114,115^ Apart from supporting the fibrillation, it has to be taken into account that mechanical performance will be diminished (Fig. 4B), and significant amounts of solvents are necessary during the process. Tailoring reactive agent quantities and reaction time can allow for maintaining low DS values where, we presume, the acetylation is confined to the surface, and enable the preparation of acetylated CNCs^116^ and CNFs.^111^ Reactive ball-milling in aprotic polar organic solvents, such as DMSO and DMF, in presence of anhydrides or acid chlorides without any additional catalysts, is surface-confined and enables energy-efficient production of hydrophobic CNF esters with variable ester length.^117–119^ Thereby wood-based CNFs were prepared with a DS of approx. 0.5; which is in the range of full surface modification of C6– and C2–OH.^43^

**Fig. 6:**
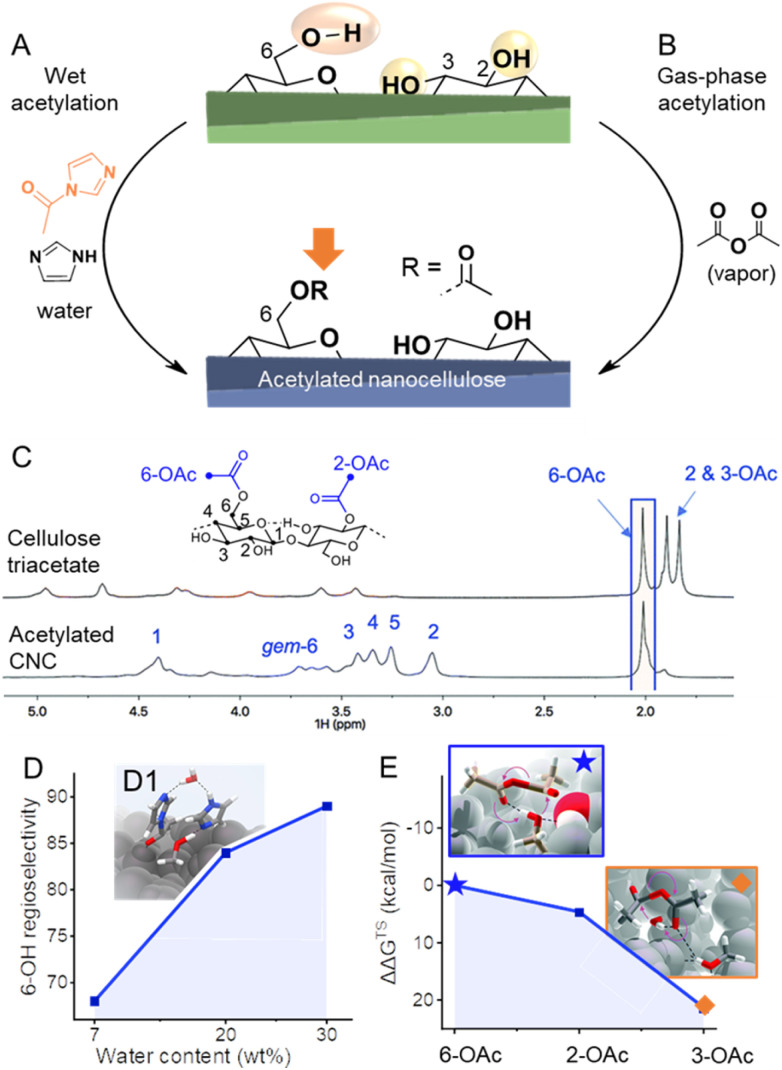
(A) Highly regioselective esterifications have been achieved through wet acetylation using *N*-acylimidazole (A) or by gas-phase acetylation with acetic anhydride (B). In both cases, selectivities arise from different accessibilities/reactivities of the OH-groups at the cellulose surface. Diffusion-edited ^1^H-NMR spectra in the ionic liquid-electrolyte [P_4444_][OAc] : DMSO-d_6_ enables the determination of the regioselectivity of the reaction, as shown in the comparison of the NMR spectra of cellulose triacetate and acetylated cellulose nanocrystals (CNC, C). In the case of wet acetylation, more water reduces the accessibility of the secondary OH-groups, increasing the regioselectivity toward C6-OH (D). Transition state modeling proves the involvement of water in wet acetylation (D_1_). Due to the significant difference in the relative Gibbs free energy of the transition states (ΔΔ*G*^TS^) of the acetylation of C6–, C2–, and C3–OH (E), mild acetylation in the gas phase favors greatly 6-OAc acetylation. Transition states of the formation of C6–(★) and C3–OAc (◆) are shown in the insets. (C) and insets in (E) were reproduced from Koso *et al.*^44^ with permission from the Royal Society of Chemistry. (D_1_) was adapted with permission from Beaumont *et al.*^43^ under CC BY 4.0. Copyright 2021 Springer Nature. Plots (D) and (E) were drawn from literature data by Beaumont *et al.*^43^ and Koso *et al.*^44^ respectively.

Not many articles are paying attention to the spatio- and regioselectivity of esterifications. The actual selectivity of a reaction is hardly determined and mainly predicted by comparing the actual DS with the theoretical one corresponding to surface modification, and by proving that the crystallinity of the sample was preserved during modification.^42^ Hence, selectivity for esterification of C6-OH *vs.* C2-OH or C3-OH was only assumable, as the most popular cellulose analysis methods often do not provide sufficient resolution. ^13^C CP MAS NMR analysis has been used to provide proof of selective C6–OH esterification,^120^ however, the resolution with this method is too limited to exclude modifications of C2–OH and C3–OH at lower DS. This is very important as it has been recently shown that in presence of a catalyst or at elevated temperatures, the acetylation is favored at C6–OH of CNC, but is not highly selective, as also modification of secondary OHs (C2–OH and C3–OH) is occurring at significant levels.^44^ The more traditional method of perpropionylation, which is used for regioselectivity determination of esterified cellulose under homogeneous conditions^121^ is also limited due to the rather low DS values in surface-modified nanocelluloses, poor signal-to-noise of the ^13^C NMR carbonyl signals, and has to the best of our knowledge not been applied for regioselectivity determination on esterified CNCs or CNFs. However, quite recently it became possible to perform high-resolution solution-state NMR on (nano)celluloses by using the ionic liquid-electrolyte [P_4444_][OAc] : DMSO-d_6_ as solvent (Fig. 6C).^43,122,123^ This has given the ability to run the more sensitive and relatively high-resolution quantitative ^1^H NMR on CNCs^44,123^ and CNFs,^43,53^ in addition to even higher resolved 2D experiments and 1D quantitative ^13^C experiments. This analytical method enabled the development of esterification methods of high regioselectivity *via* modification of cellulose either in solid, suspension, or gas states. The reaction of *N*-acetylimidazole enables modification of C6–OH at high selectivity, can be conducted in a solid state, and is promoted by confined water in the hydration layer of cellulose fibers (Fig. 6A and D_1_).^43^ The high efficiency of this reaction was explained by the fact that the reaction takes place in the confined water layer of cellulose, which covers all microfibril surfaces. The results indicate that these water clusters are connected continuously or allow at least the diffusion of solubilized reactant along internal fibrils. Moreover, it has been shown that increasing the thickness of this layer increases the regioselectivity towards C6–OH of cellulose and enables thereby highly selective reactions (Fig. 6D). This system can be also extended to the preparation of functional CNFs, cellulose fibers have been in this case directly modified with *N*-acylimidazoles in a mixture of water and acetone.^43,48,53^ The *N*-acylimidazoles can be either directly added or prepared *in situ* from the corresponding carboxylic acid anhydrides and imidazole. Upon esterification, the functional fibers were fibrillated to yield CNFs decorated with either acetyl, *iso*-butyl,^53^ or succinate groups.^90^ Introduction of negative charge *via* succinate groups enabled complete individualization (through increased electrostatic repulsion) of succinylated CNFs,^90^ comparable to the well-known TEMPO-oxidized CNFs.^124^

Apart from esterifications with acylimidazoles in presence of water, gas-phase acetylation of CNCs with acetic anhydride is highly regioselective for the surface C6–OH positions (Fig. 6B).^44^ Further to this, a comparison between the uncatalyzed gas- and liquid-phase reactions yielded similar reaction kinetics. However, the gas-phase reaction was found to be more regioselective, supporting the concept that the correct choice of solvent or complete absence of solvent is required to yield high C6–OH regioselectivity. Moreover, density functional theory transition-state modeling on cellulose I fragment demonstrated that the activation energies for esterification of the three different OH groups using acetic anhydride are rather different, with the C6–OH being much more favored, which explains the high regioselectivity under mild gas-phase conditions (Fig. 6E). The CNC morphology was found unaffected by the gas-phase acetylation, whereas it was noted that the fully acetylated surfaces of CNCs can be peeled off in dipolar aprotic organic solvents, such as DMSO, which is also visible in Fig. 6.^44^ Similar observation was also made for TEMPO-oxidized cellulose,^14^ and is important to take into account that in case of C6–OAc–CNC, it only occurs in special organic solvents, and not in water, which is the standard solvent. When the liquid-phase reactions were catalyzed using organic bases pyridine and DABCO, a progressive conversion of the outer CNC surfaces, to CTA, towards the core was observed. Based on this knowledge one would expect that under mild and non-swelling conditions it should be possible to control the reaction to only surface C6–OHs. Nevertheless, the reactivity of the acylation agent and temperature are also important, *e.g.*, gas-phase reactions of acid chlorides are non-surface-selective and proceed also in the core of CNC,^120,125^ whereas surface-selectivity was achieved when dicarboxylic acid chlorides are used, such as suberoyl chloride.^126^

The mechanistic understanding of the surface chemistry of CNCs and CNFs is developing to a point where the confinement of reactions to nanocellulose surfaces can be controlled. Further studies of the regioselectivity of various cellulose modifications are expected to lead to the establishment of more advanced and versatile methods for chemical modification, which do not have to be limited to esterification reactions but could also encompass other substitution reactions, such as etherifications^127^ or sulfonation.^128^

#### 3.3.2 Oxidations (C2–, C3–OHs, or C6–OH selectivities)

Chemical and enzymatic oxidations are established for cellulose with the aim of oxidizing selected hydroxyl groups, to aldehydes and carboxylic groups, or *via* hydrolysis and oxidization of the glycosidic bond. In the case of enzymatic oxidation with, *e.g.*, lytic polysaccharide monooxygenases (LPMOs),^129,130^ oxidation is coupled with simultaneous cleavage of the glycosidic bond, usually at the C_1_ and C_4_ positions.^129^ The cleavage and oxidation lead to products of oxidized solubilized molecules or oxidized cellulose substrates.^131^ Enzymatic modifications are hence both hydrolyzing and functionalizing and can be applied to the various cellulose hierarchies. However, when it comes to the oxidative function, the balance between liberating functionalized fractions from the solid substrate and preserving the functionality of (nano)celluloses is still to be developed, especially when the latter is the target.

**Fig. 7:**
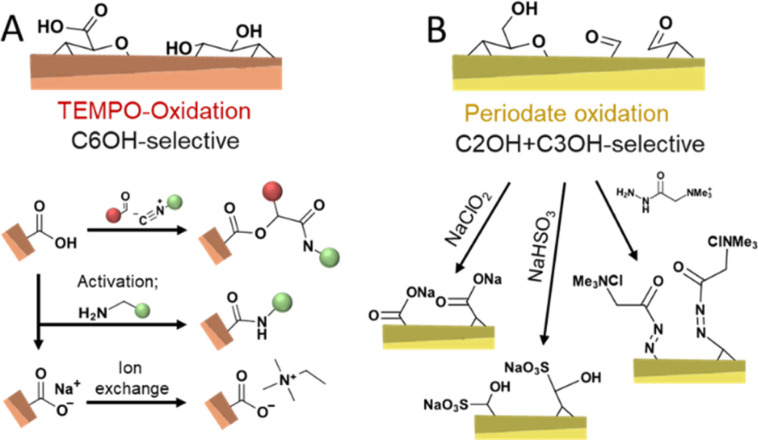
Oxidation of cellulose by TEMPO-(A, orange colored) and periodate-oxidation (B, yellow-colored) introduces selectively carboxyl moieties at C6 or aldehyde groups at C2 and C3, respectively; enabling a myriad of follow-up chemistries.

Chemical routes of oxidizing cellulose are dominated by TEMPO-mediated (Fig. 7A)^11,124,132,133^ and periodate oxidation (Fig. 7B)^75,104,134–136^ as well as their combination.^137,138^ TEMPO-oxidation is considered selective to oxidize the primary hydroxyl group at the C6 position and proceeds through the *N*-oxoammonium ion formation from TEMPO radical, which converts alcohol into aldehyde, followed by its further oxidation to carboxylic acid by oxidant, usually, hypochlorite.^11^ When the aim of a modification *via* TEMPO-oxidation is to decorate nanocellulose with carboxylic groups at the C6 position, the oxidation can be performed on cellulose fibers before disintegration into CNFs, or on the already liberated CNFs. Oxidation of cellulose fibers leads to significant swelling and loosening of the fibril networks^133^ and hence, aids the fibrillation process.

Periodate oxidation proceeds by the reaction with the C2–C3 diol, cleavage of this bond, and conversion to two aldehyde groups that can further form hydrates, hemialdals, and, with vicinal hydroxyl groups, hemiacetals.^135,139^ Both TEMPO and periodate routes to modify cellulose are considered as regioselective reactions with regards to the position in the monomer unit, also referred to as anhydroglucose unit (AGU), that can be oxidized. It is important to consider that these oxidative conditions usually cause a decrease in the molar mass of the cellulose.^140,141^ Nevertheless, TEMPO-mediated oxidation is commonly selected when surface modification of cellulose^34^ is targeted and very popular to individualize cellulose fibers into high-performance TEMPO-oxidized CNF (TO-CNF).^142^ As discussed in Section 3.2, CNCs are conventionally prepared through acid hydrolysis to further deconstruct cellulose's elementary fibrils, alternatively, cavitation treatment of TO-CNFs enables an acid-free preparation of CNCs.^143^ TEMPO-oxidation seems to destabilize to a certain extent further defects or weaknesses of CNFs, which would be otherwise attacked in the typical hydrolytic deconstruction. TEMPO-oxidation has been also used to modify CNCs obtained through HCl hydrolysis, which would be otherwise unstable due to their low surface charge.^144^ In addition, the carboxylate groups introduced through TEMPO-oxidation can also be used to introduce further functionality through amidation reactions,^145^ by ion-exchange of Na^+^ with ammonium salts,^146,147^ or through a three-component Passerini reaction (Fig. 7A).^148^

The periodate oxidation, on the other hand, is principally considered as not highly selective to accessible surfaces and propagates also into crystalline domains.^140,149^ However, this is still under debate since recent approaches report reactions that can be controlled to attack preferentially the less ordered regions.^91^ Revelation of the simultaneous but different rates of oxidations on the ordered and disordered regions are evaluated to understand the oxidation fundamentals and to allow them for controlled functionalization purposes. When functionalization of CNCs or CNF is targeted, taking the degradation into account, oxidation of cellulose needs to be controlled to limit its influence on the physical properties, similar to what has been achieved with the wet esterification method using acylimidazole that enable highly regioselective esterification of the C6–OH of cellulose while preserving the cellulose molecular weight and crystallinity.^43,48^ Controlling the degree of oxidation to a range of accessible surface hydroxyl groups enables the preparation of celluloses decorated with aldehyde groups, which can be post-modified to yield C2, C3-carboxylated,^104,152^ sulfonated,^153^ or cationic CNFs in a straightforward manner (Fig. 7B).^154^ These repulsive charges support, similar to carboxylate groups, the fibrillation of the fibers into individualized CNFs. Apart from these reactions, the reactivity of aldehyde enables the introduction of a plethora of functional groups.^1,155^ Mild periodate oxidation of CNCs and further oxidation to carboxylic groups with ozone have been demonstrated to cellulose Janus films with side-specific chemical functionality (aldehyde *vs.* carboxylic acid).^136^ Periodate oxidation to a higher extent is an efficient way of modulating cellulose hierarchies,^135^ and leads, *e.g.*, to the formation of dangling polymer ends attached to the CNCs, so-called hairy nanocrystals.^156,157^ Moreover, it allows the direct preparation of CNCs from micron-sized cellulose or biomass,^158^ even in complex systems, such as Pickering emulsions (Fig. 6D).^159^ Combined TEMPO and periodate oxidation has been used to produce highly carboxylated and charged nanocellulose,^160^ which can self-fibrillate into CNFs as a response to change in pH.^133^

All in all, both periodate^135^ and TEMPO-oxidations^11,34,124^ have been reported to cause polymer degradation for oxidation degrees in the range of surface modifications. Although this influence on molar mass can be reduced under mild conditions, *e.g.*, through TEMPO-oxidations at neutral/acidic pH,^161,162^ it still limits the possible mechanical properties of oxidized CNCs and CNFs.^1,49^ In addition, it is important to take into account that treatment of cellulose with such oxidizing agents might as well cause side reactions, *e.g.*, the oxidation of secondary hydroxyl groups. In the case of TEMPO-oxidation, post-oxidation is necessary to oxidize the remaining C6-aldehyde groups.^124^ Moreover, occurring reactions are getting rather complex in presence of lignocelluloses, *e.g.*, periodate degrades lignin and also oxidizes hemicelluloses, such as xylan.^163^ This might be a desired side-effect, but reduces the efficiency of oxidations, and does not allow a selective modification of individual lignocellulosic polymers. In addition, the recycling of periodate is possible but requires a rather complex setup.^164^

While TEMPO-oxidation has been recognized to be highly selective to the surface of cellulose fibrils without affecting the crystallinity of cellulose,^142,143^ this is still under debate in the case of periodate oxidation, but unwanted reactions of crystalline, non-accessible regions can at least be kept to a minimum if reaction conditions are carefully chosen.^135^ Consideration of the high efficiency of such oxidations, the fact that they are compatible with water as the solvent, and the many possibilities for post-modifications, explain the high potential of these reactions and why they are widely used in the preparation of nanocelluloses.

#### 3.3.3 Spatioselective modification of reducing end-groups

End-group modifications are conducted exclusively on CNCs, which is due to (a) their lower length and hence the more dominant influence of the end-group functionality on CNC properties, and (b) the spatial location of the chain end-groups at one side of the CNC.^165^ However, the general labeling of end-groups can be also performed on fibers or CNFs, and is performed, *e.g.*, for analytical studies;^168^ but their locations are not as defined as in the case of CNCs (as those might be situated as well along the fiber axis and not only at their end).

**Fig. 8:**
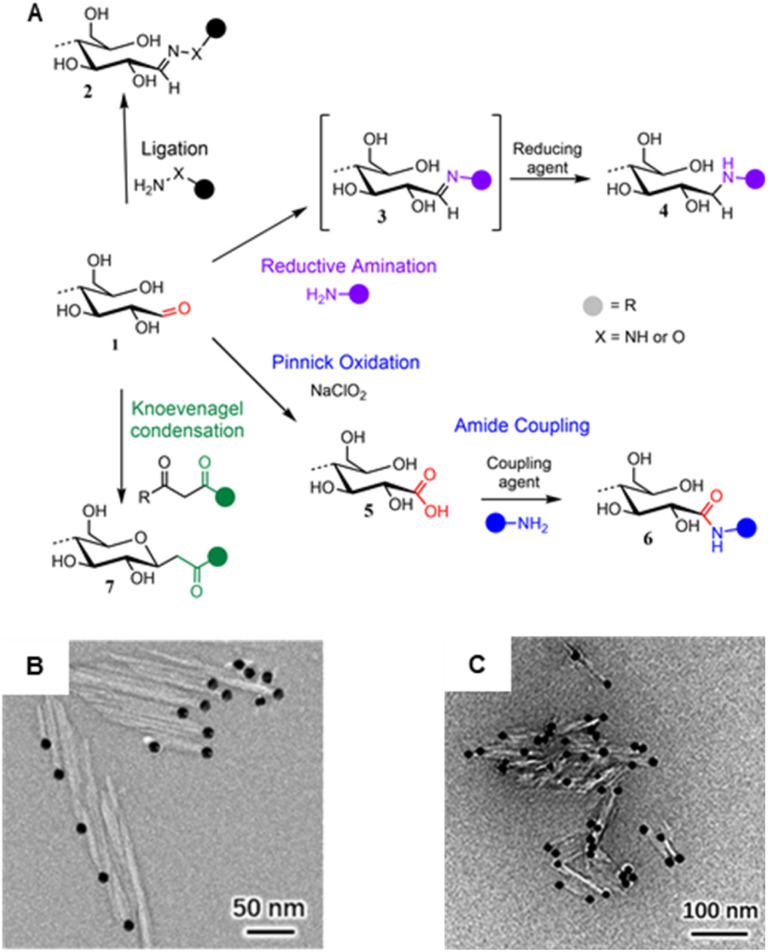
(A) Main mechanisms used to target cellulose REGs (1): Ligation of hydrazine (NH_2_–NH–R) or hydroxyl amine derivatives (NH_2_–O–R) affording hydrazones or oximes (2); reductive amination forming an imine intermediate (3) that is reduced to a stable secondary amine (4); Pinnick oxidation affording carboxylated REGs (5) that allow for subsequent amidation (6); Knoevenagel condensation affording a C-glycoside ketone (7). (B) Asymmetric thioureation of the reducing ends of CNCs during which the aldehyde groups are converted into imine groups, and TEM images of CNC (left) and CNC-II (right) are modified with gold nanoparticles. (A) was adapted with permission from Heise *et al.*^165^ under CC BY 4.0. Copyright 2020 Wiley. (B) was reprinted from Lin *et al.*,^166^ Copyright 2021, with permission from Elsevier. (C) was reproduced from Lin *et al.*^167^ with permission from Royal Society of Chemistry.

Despite the increasing variety of functionalities being grafted to CNC REGs, the basic chemistry used to approach the end-standing aldehyde groups is not very broad with only four prevailing mechanisms (Fig. 8A): (1) ligation of hydrazine analogous,^169–173^ hydroxylamines,^174^ or thiosemicarbazide,^166^ (2) Pinnick oxidation forming carboxyl groups,^175–178^ (3) reductive amination attaching primary amines,^179–184^ and (4) Knoevenagel condensation with dicarbonyls.^174^ The peculiarities and the different reaction conditions for each of these mechanisms are described in two recent reviews and will, therefore, not be discussed in detail here.^185,186^

Given the nature of CNCs, including their excellent colloidal stability in water, most reactions are carried out in an aqueous environment. This also aids the activation of the REGs as the reactive open-ring aldehyde is in equilibrium with the non-reactive hemiacetal form. In theory, one water molecule^187^ should be enough to sufficiently catalyze the proton transfer opening the REG hemiacetal and liberating the reactive aldehyde. However, to further shift the equilibrium to the aldehyde, mildly acidic (*i.e.*, pH 4.5)^181,182^ or alkaline (*e.g.*, aqueous bicarbonate at pH 8.5)^174^ conditions have been used. In the absence of water, the ring opening can be catalyzed by a mixture of organic acids and bases acting synergistically in a concerted mechanism to enable proton transfer at the REGs.^188^ On CNCs, these catalytic possibilities are yet to be explored.^189^

Besides the pH and solvent conditions, the reaction temperature, affecting the mutarotation rate,^190–192^ and the reaction time are relevant for the cellulose end-group conversion. For sufficient conversion rates, the reaction times are usually very long, easily exceeding 48 hours, which might be a major challenge when up-scaling CNC REG-modification to industrial applicability. This limitation, however, might be surrendered with more efficient catalysis.

Especially in the realm of synthesizing Janus-type nanorods from CNCs, the aldehyde-specific modification of the REGs is only the initial, activating step for introducing, *e.g.*, high-molecular-weight compounds like polymers, dendrimers, metal nanoparticles (Fig. 8B) or biomolecules to the CNCs. In general, one has to distinguish between native CNCs of cellulose I crystal structure or CNCs obtained through mercerization with NaOH, which changes their crystal structure into cellulose II (CNC-II). Dependent on the nature of CNCs, end-groups are either located anisotropically at one (CNC) or both ends (CNC-II). This difference is visualized using TEM and CNCs with metal nanoparticle-labeled end-groups (Fig. 8B).

Grafting-to approaches are very common in the modification of CNCs, where 1-ethyl-3-(3-dimethylaminopropyl)carbodiimide and *N*-hydroxysuccinimide (EDC/NHS)-mediated amidation to previously oxidized REGs (*i.e.*, *via* NaOCl_2_ Pinnick oxidation) is probably the most popular pathway.^175–178,180^ This also highlights that the first aldehyde-specific step on the pristine REGs determines the selectivity of the later grafting protocols. Zoppe *et al.*, for instance, observed side reactions on the CNC surface after oxidizing the REGs with sodium chlorite followed by grafting polymerization initiators, using a two-step protocol, for subsequent atom-transfer-radical polymerization.^175,177^ The result was a patchy distribution of polymer chains rather than a Janus-type architecture. The reason for this side reaction could be impurities of oxidized moieties on the CNC surface or defects in the nanocrystal structure, *i.e.*, REGs that are situated at the nanocrystal surface and not exclusively at the end. In general, we have to remember that we are working with naturally sourced nanomaterials bearing both the imperfections of their top–down isolation (*e.g.*, impurities, surface roughness) and heterogeneous size- and molecular-weight distribution.

Side-reactions during the REG modification can also negatively affect the later polymer graft density, as shown in a recent contribution by Delepierre *et al.*^181^ They observed that significant proportions of the amino-functionalized initiator were passivated during its attachment to the REGs *via* a reductive amination protocol, at 70 °C and pH 4.5, in a 72 hours reaction. This again highlights how challenging especially multi-step protocols at REGs can be and how important it is to thoroughly understand and select the chemistry. In terms of selectivity, the use of enzymes acting specifically at the reducing or non-reducing ends might be a promising and green future avenue toward a Janus-type CNC modification – a concept that is, to the best of our knowledge, still out of reach for modifying nanocelluloses.

### 3.4. Analysis of spatioselectively modified nanocelluloses

Spatial analysis of various functionalities on cellulose hierarchies is challenging and often requires a combination of techniques delivering qualitative and, if needed, quantitative (DS) information. Visualization and mapping of functionalities spatially in particle hierarchies, such as nanocelluloses, usually requires microscopy and the introduction of bulky functionalities with sufficient contrast, or microscopy combined with spectroscopic techniques. Atomic force microscopy (AFM) is superior for the visualization of morphologies. It also allows the identification of chemical features on surfaces indirectly *via* adhesion^193^ or directly *via* employing functionalized tips^194–196^ that have a different affinity to the diverse chemical functionalities on the surface and this can be quantified by measurement of attractive or repulsive force. However, one of the bottlenecks of AFM techniques is the dimensions of the tips defining spatial resolution. For circumventing this, extensions to the traditional AFM techniques are developed.^197^ Combining AFM with IR spectroscopy, for example, has been used for the mapping of wood cell wall components with nanometer resolution based on differences in chemical composition.^198^ In addition, it is to be expected that further development of scanning electron microscopy (SEM) and transmission electron microscopy (TEM) methods coupled with energy-dispersive X-ray spectroscopy (EDX) will be able to reveal further details of the microfibril chemistry and allow more detailed chemical mapping. The main obstacles of electron microscopy are the limitations of electron beam radiation to low voltage, to avoid changes in the surface morphology and chemistry of the samples, which limits the resolution of this method.^199^ For instance, progresses in SEM-EDX, which allows localized elemental analysis, are expected to increase in resolution, once the beam damage of the specimen can be reduced or avoided.

**Fig. 9:**
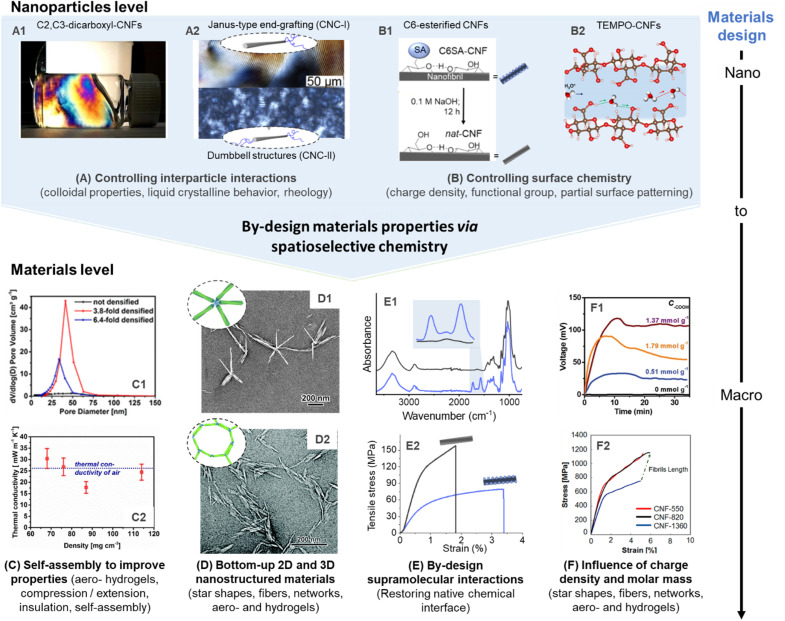
Influence of spatioselective modification of nanocelluloses on nanoparticle and materials properties. C2, C3–dicarboxyl–CNFs (produced by sequential periodate and Pinnick oxidation) forms highly ordered CNF dispersions (A_1_), which can be processed into aerogels with controlled porosity and alignment (C_1_) to enable optimal thermal insulation behavior (C_2_). Anisotropic and isotropic end-group grafting of CNCs can be used to influence their order in liquid crystalline films (A_2_), and enable self-assembly into star-shaped (D_1_) or network-like superstructures (D_2_). Reversible succinylation at the C6–OH enables individualization into CNFs and is reversible to restore the native chemical interface of cellulose (B_1_). This can be used to produce highly stiff nanopapers of assembled native CNFs (E_1_ and E_2_). TEMPO-CNFs and their regularly patterned carboxyl moieties enable, *e.g.*, high ion conductivity (B_2_), and allow straight-forward tuning of their degree of oxidation for optimal electricity generation (F_1_), as well as to limit polymer degradation for the production of ultra-strong fibers (F_2_). (A_1_), (C_1_), and (C_2_) were adapted with permission from Plappert *et al.*^104^ Copyright 2017 American Chemical Society. (A_2_) was adapted with permission from Delepierre *et al.*^182^ Copyright 2021 American Chemical Society. (D_1_) was adapted with permission from Lin *et al.*^200^ Copyright 2019 American Chemical Society. (D_2_) was reproduced from Lin *et al.*^167^ with permission from Royal Society of Chemistry. (B_1_), (E_1_), and (E_2_) were adapted with permission from Beaumont *et al.*^90^ under CC BY 4.0. Copyright 2021 American Chemical Society. (B_2_) was adapted with permission from Bayer *et al.*^215^ Copyright 2017 American Chemical Society. (F_1_) was reprinted with permission from Li *et al.*^216^ Copyright 2019 Wiley. (F_2_) was reprinted with permission from Mittal *et al.*^217^ Copyright 2018 American Chemical Society.‡‡Further permission requests related to the material excerpted should be directed to the American Chemical Society.

Alternatively, the self-assembly behavior of spatioselectively modified nanocelluloses, as in the case of end-group functionalized CNCs (Fig. 9B_1_–B_3_) has been used to visualize and confirm selective chemical modification by microscopic means. For instance, star-shaped CNC assemblies (Fig. 9B_2_), formed in response to increased temperatures^200^ or through end-wise crosslinking,^178^ have been visualized by AFM and TEM. Also, labeling with metal nanoparticles (Fig. 8B) is a well-established way to visualize functionalized REGs of CNCs.^166^

For simultaneous visualization of structure and chemistry, the combination of microscopic and spectroscopic techniques is advantageous. Imamura *et al.* combined microscopy with FTIR spectroscopy and visualized carbonyl functionalities on cellulose fibers.^201^ However, the resolution of the microscopy still limits the resolution of the observation, and reaching the nanoscale is a challenge. CNC ordering in suspension and solids enables structural color and tuning of mechanical and optical properties. Kádár *et al.*^202^ reviewed the combination of rheology methods with other techniques for revealing chiral nematic ordering; and combining rheology that probes macroproperties with for example X-ray analytics^203^ is not far away from reaching visualization resolution of an individual CNCs. The combination of macroscopic, microscopic, and atomic methods in line would enable *in situ* analytics where spatial observations can be related to sample interactions during processing.

High-resolution solution-state 1D and 2D NMR techniques have molecular resolution and can be used to confirm spatioselective modification, as already mentioned above. The accessibility of solution-state NMR to nanocelluloses is not self-evident since crystalline celluloses do not dissolve in typical perdeuterated solvents. Tackling this bottleneck, King and co-workers have developed NMR methods that use the ionic liquid electrolyte [P_4444_][OAc] : DMSO-*d*_6_ to dissolve nanocelluloses.^204^ This ground-breaking development has, for instance, enabled in-depth qualitative and semi-quantitative analysis of REG-modified CNCs (Fig. 8),^174,181^ oxidized^122^ or regioselectively esterified^205^ nanocelluloses. In addition, this method can also take advantage of existing structural assignments to yield detailed regioselectivity information,^122,123,206–210^ to give insights on regioselectivity on a variety of chemical modifications.

As another method of delivering molecular information, X-ray photoelectron spectroscopy (XPS) and, specifically, the high-resolution carbon (C 1s) region can be used to differentiate, *e.g.*, O–C=O, O–C–O, C–O, and C–C linkages of oxidized celluloses.^211^ However, the limited analysis depth of XPS to < 10 nm restricts this method to the surfaces,^212^ strictly defining requirements for the sample preparation to obtain reliable information. In addition, it is so far not possible to distinguish a substitution at the different OH-groups of cellulose to prove for example, a regioselective modification. Moreover, experimental factors, including contamination before and outgassing or degradation of the sample during the measurement may bias the XPS data.^211^

For reliable quantitative analysis of spatioselectively modified nanocellulose, *i.e.*, for determining the surface DS, the range of different, especially, wet-chemical methods is broad, including the classical charge titration for determining surface carboxylates, infrared spectroscopy,^43^ or derivatization of surface groups and their later quantification by fluorescence or UV spectroscopy.^174,213,214^ In the case of REGs, there are multiple ways to determine the number of REGs. One possibility is the utilization of copper complexes (*e.g.*, the Cu I bicinchoninate complex) coupled with spectrophotometric quantification.^175,181^

## 4. Implications of spatioselective nanocelluloses on materials science

The impact of spatioselective modifications of cellulose in biomass is multifold. At the building block scale, it enables the extraction of the elementary fibrils of cellulose, the control over colloidal stability, and ion interactions. Moreover, interfacial interactions can be finely tuned by changing the spatial distribution of functional groups, affecting as well their affinity for self-assembly and adhesive interactions with non-cellulosic interfaces. Finally, a selective modification will highly influence the mechanics of the obtained nanocellulosic material and their optical properties as associated with the long-range order of the obtained fibers. We showcase below some relevant examples.

### 4.1. Controlling interparticle interactions

For manufacturing of CNFs, TEMPO-mediated, and periodate oxidation are currently pivotal for controlling the colloidal properties. The introduction of charged groups facilitates electrostatic repulsion that aids colloidal stability. Regioselectively modified CNFs have been shown to possess extraordinary long-range ordering capability, even at low concentrations below 1 wt% in water, and yield anisotropic alignments with liquid crystalline behavior (Fig. 9A_1_).^104,218^ The high ordering capability of these CNFs can be attributed to their regular patterning of functional groups, *e.g.*, in the case of C2, C3–dicarboxyl-CNFs, or TEMPO-CNFs. This structure can be also translated into ordered and mechanically robust aerogels, and enable strain hardening and pore size harmonization (Fig. 9A_2_) by compression-induced alignment to reach optimal heat insulation properties (Fig. 9A_3_). Other properties may be optimized with such considerations, for instance, the gas permeability that is a key parameter for cellulosic to form sustainable replacements for single-use plastics used as food packaging. Noteworthy, completely individualized CNFs have been obtained so far only from cellulose modified with self-repulsive charges (*e.g*., carboxylates, sulfonates, *etc*.), and the preparation of completely individualized hydrophobic CNFs is still a challenging task, especially using spatioselective chemistry, which does not partially solubilize or degrade the cellulose sample. Concerning CNCs, self-assembly and alignment play even a more dominant role and gives access to films or coatings with structural color,^219,220^ or allows the expansion of the upper limits in mechanical properties of CNC materials.^221,222^ So far, most related efforts have been conducted with CNCs obtained from sulphuric acid hydrolysis, which have a small number of sulfate groups at their surface.

Colloidal properties can be also controlled by spatioselective modification of the CNC end groups. Polymer grafting is one concept that has been described for CNC and CNC-II and that offers two main design parameters for tuning the properties of the semi-synthetic nanohybrid: the physicochemical nature of the polymer chains and their molecular weight. The grafting of hydrophobic polystyrene from the REGs of CNCs, for instance, renders the particles amphiphilic and has improved their ability to stabilize oil-in-water emulsions.^183^ Similar concepts might be also interesting for the compatibilization of immiscible polymer blends^223^ while simultaneously enhancing mechanical properties and the dispersibility of the CNCs in the polymer matrix. The grafting of hydrophilic polymers, on the other hand, has been described to improve the redispersibility of previously dried CNCs in aqueous media,^181,182,224^ while maintaining, for instance, their ability to form liquid crystalline phases (Fig. 9A_2_).^182,224^ This has been shown for asymmetrically or symmetrically grafted CNCs with thermo-responsive polymers at their end-groups enabling the formation of chiral nematic tactoids.^182^ This behavior was especially interesting for the smaller CNC-II, where the polymer grafting introduced long-range orientational order and allowed the CNC-II to self-assemble 40-times faster than its unmodified counterpart. Jean and co-workers grafted temperature-responsive Jeffamine polyether amines to the end-groups of CNC and CNC-II (Fig. 9B_1_) to control their assembly into defined superstructures.^167,225^ The star shapes (Fig. 9B_2_), observed for end-grafted CNC-I above the LCST of the grafted polymer, are an impressive example of controllability and reproducibility of CNC self-assembly.^225^ CNC-II, symmetrically grafted with Jeffamine chains, formed micron-sized networks of end-to-end connected CNC-II grafts (Fig. 9B_3_).^167^ These complex 2D and 3D nanostructures cannot be achieved through uniform surface modification of CNCs.

Apart from CNC-II, there has been also a recent effort in the preparation and characterization of soft cellulose II nanospheres.^226,227^ They have unique swelling capability due to their less crystalline particle shell,^227^ which can be also used to enable the fabrication of surfaces with high protein loading for improved detection sensitivity,^228^*e.g.*, in COVID-19 antigen tests.^229^ Details on their exact morphology and structure are still under debate, and although regioselective modification has not been adopted on this type of nanoparticles, it might support improving their properties and establishing a better understanding of cellulose chain assemblies in different types of nanocelluloses.

### 4.2. Controlling surface chemistry

Apart from controlling self-assembly, selective surface modification can be also further tuned to vary, *e.g.*, the type of functional group or the degree of surface modification. Typically, the interactions occurring at cellulose interfaces will significantly impact the mechanical properties but also the adsorption isotherms, binding constants, and adsorbed conformation of other macromolecules (hemicellulose, lignin, proteins, *etc*. in non-processed cellulosic fibers) and molecules (*e.g.*, surfactants, ions, *etc.*).^230–232^ In turn, beyond controlled mechanics, a range of key properties will be associated with these parameters such as water interactions,^216,233^ biological responses,^234,235^ colloidal stability,^236,237^ ion interactions (*e.g.*, conductivity),^238,239^ or enzymatic activity.^240,241^ These interactions of nanocelluloses can be tuned by varying, *e.g.*, the type of functional group and the degree of surface modification. C6–O–succinylated–CNF has been shown to possess similar properties as TEMPO-CNF but offers apart from its higher molar mass another clear advantage as the ester linkage in C6–O–succinylated–CNFs can be cleaved to recover the intrinsic cellulose chemical interfaces (Fig. 9C_1_).^90^ This was also demonstrated at the materials level, nanopapers prepared from C6–O–succinylated–CNFs were treated under alkaline conditions to remove/hydrolyze the ester groups (Fig. 9C_2_). Thereby, hydrogen bonding and fibrillar interactions can be strengthened significantly (Fig. 9C_3_), increasing 2.5-fold the tensile strength and reaching an elastic modulus of 19.4 GPa (in comparison to 7.9 GPa before ester hydrolysis).

The extent of a C6–O-carboxylation of CNFs can be modulated by varying, *e.g.*, the reaction time of the TEMPO-oxidation.^11^ A higher degree of C6–carboxyls introduced goes hand in hand with a lower molar mass,^11,141^ as well as stronger repulsive interactions and water interactions, which may limit the performance of CNF assemblies.^216^ Based on this fact, there is generally an optimal degree of oxidation, which is below complete surface oxidation, as shown in the dependence of the open-circuit voltage of TEMPO-CNF aerogels on the charge density of CNFs (Fig. 9D_2_).^216^ This was also well demonstrated during the preparation of highly aligned, ultra-strong cellulose microfibers (Fig. 9D_3_).^217^ In the former case, CNFs with the highest carboxylate content produced less energy, explained by over hydration and structural collapse of respective aerogels,^216^ whereas in the latter case the lower mechanical stability of highly charged CNFs is explained by their lower degree of polymerization.^217^ Taking this into account, alternative preparation of CNFs, *e.g.*, through selective succinylation, are noteworthy to be investigated and could potentially increase the performance of assemblies of charged CNFs, due to their higher degree of polymerization. Surface modification of CNCs by TEMPO-oxidation has been also shown to induce synergistic interactions in composites, as shown in the case of polypyrrole/CNC films.^242^ In this case, it was demonstrated that the carboxylate groups at the CNC surface, increased aerial capacitance, energy density, and cycling stability in prepared supercapacitors.

Dependent on the application of the material, the use of mild gas-phase reactions can be of interest for post-processing, *e.g.*, hydrophobization, to avoid the occurrence of swelling and/or capillary forces during the reaction or the drying step, which would occur in liquid state reactions. The C6–OH selective gas-phase acetylation was used to modify and thereby hydrophobize labile and complex chiral-nematic aerogels.^44^ Regular treatment in organic solvents would disrupt this delicate and structurally colored assembly. This treatment can be applied also to other labile materials or potentially be used to produce regioselectively modified CNC model films for fundamental studies.

## 5. Conclusions

Considering the defined structure of CNCs and CNFs, and the possibility to regularly pattern functional groups at their surface, there are many hidden opportunities in the chemistry of nanocellulosic materials. In this review, we summarized basic knowledge in this field to give the reader the toolbox and background information to prepare such spatioselectively modified nanocelluloses, as well as to demonstrate its long-term potential in materials science.

Most focus on spatioselective modification has been set on endwise modification of CNCs, which was proven to significantly alter the colloidal properties, as well as the interactions of CNCs with themselves and other polymers/particles, in the nano- and materials scale.

Concerning modification of the surface hydroxyls, it has been clearly shown that a restriction to a surface modification preserving crystalline, non-accessible cellulose regions, is crucial to improve the properties of nanocellulosic materials, such as transparency, mechanical and thermal properties. In addition, although clear evidence is rare and typically unavailable, there are indications that a regioselective patterning increases the tendency of nanoparticles to align and improve the mechanical properties of their assemblies. For example, in the case of TEMPO-oxidation, it is unclear if the influence of carboxylate content on CNF properties is dominated by charge density, molar mass, hydration effects, or a combination of these. This is a good demonstration of how complex it is to give a clear statement on the role of selective patterning of nanocelluloses.

Moreover, there is yet a lack of analytical and non-invasive methods to analyze the regioselectivity of nanocellulose modifications and to visualize a certain spatial distribution directly at the surface of those nanoparticles. Recent developments in the chemical analysis of nanocelluloses enabled direct determination of the regioselectivity of esterified CNFs and CNCs through high-resolution NMR spectroscopy in an ionic liquid electrolyte. Based on this method, regioselective pathways using *N*-acylimidazoles or gaseous acetic anhydride were established, which are surface-selective, and versatile (as different types of ester groups can be introduced). Initial research has already demonstrated their potential to surpass current limitations, *e.g.*, in the mechanical properties of nanocelluloses. In this case, the motivation is clear, since the mechanical strength of nanocellulosic materials has a current maximum of approx. 1 GPa,^217,243^ and is still far from the maximum theoretical value that ranges between 2 and 7 GPa.^244,245^

There are still limitations in the available toolbox of spatioselective modifications in terms of reaction efficiency, available chemistries, and slow reaction kinetics, *e.g.*, in the case of endwise modification of CNCs. The sustainability of nanocellulose modifications is currently given little attention, many reactions use a massive excess of reactants and solvents. In addition, the atom economy of reactions and the recyclability of reactants are often not considered, rendering chemical modifications of renewable nanomaterials still problematic from an ecological and economical viewpoint.

Before functionalization, one should carefully consider the cellulosic precursor to be reacted (*e.g.*, lignocellulosic fibers, containing lignin and hemicelluloses, *vs.* cellulose fiber). Most selective reactions are conducted using highly pure cellulose fibers, which limits their feedstock to a low percentage of available biomass. Further development should also consider raw or more complex biomass, to enable selective modification of cellulose in presence of other natural building blocks. These selective modifications have also the potential to ease the deconstruction of biomass into nanocellulose, which would be important to enable a more efficient and sustainable production on a bigger scale.

Apart from the huge potential of spatioselective chemistry to fine–tune interactions and surface properties of nanocelluloses, the development of new selective pathways of high atom efficiency economy will also tremendously increase the sustainability and efficiency of chemical reactions on nanocellulosic substrates, which is especially relevant in establishing functional nanocellulose as the future building block for high-performance materials.

## Conflicts of interest

There are no conflicts to declare.

## Supplementary Material
